# Hyper-parameter tuning and feature extraction for asynchronous action detection from sub-thalamic nucleus local field potentials

**DOI:** 10.3389/fnhum.2023.1111590

**Published:** 2023-05-24

**Authors:** Thomas Martineau, Shenghong He, Ravi Vaidyanathan, Huiling Tan

**Affiliations:** ^1^Biomechatronics Group, Department of Mechanical Engineering, Imperial College London, London, United Kingdom; ^2^Medical Research Council Brain Network Dynamics Unit, University of Oxford, Oxford, United Kingdom; ^3^Nuffield Department of Clinical Neurosciences, University of Oxford, Oxford, United Kingdom; ^4^UK Dementia Research Institute-Care Research and Technology, Imperial College London, London, United Kingdom

**Keywords:** Bayesian optimization (BO), deep brain stimulation (DBS), time-frequency analysis, local field potentials (LFPs), brain-computer interface (BCI)

## Abstract

**Introduction:**

Decoding brain states from subcortical local field potentials (LFPs) indicative of activities such as voluntary movement, tremor, or sleep stages, holds significant potential in treating neurodegenerative disorders and offers new paradigms in brain-computer interface (BCI). Identified states can serve as control signals in coupled human-machine systems, e.g., to regulate deep brain stimulation (DBS) therapy or control prosthetic limbs. However, the behavior, performance, and efficiency of LFP decoders depend on an array of design and calibration settings encapsulated into a single set of hyper-parameters. Although methods exist to tune hyper-parameters automatically, decoders are typically found through exhaustive trial-and-error, manual search, and intuitive experience.

**Methods:**

This study introduces a Bayesian optimization (BO) approach to hyper-parameter tuning, applicable through feature extraction, channel selection, classification, and stage transition stages of the entire decoding pipeline. The optimization method is compared with five real-time feature extraction methods paired with four classifiers to decode voluntary movement asynchronously based on LFPs recorded with DBS electrodes implanted in the subthalamic nucleus of Parkinson’s disease patients.

**Results:**

Detection performance, measured as the geometric mean between classifier specificity and sensitivity, is automatically optimized. BO demonstrates improved decoding performance from initial parameter setting across all methods. The best decoders achieve a maximum performance of 0.74 ± 0.06 (mean ± SD across all participants) sensitivity-specificity geometric mean. In addition, parameter relevance is determined using the BO surrogate models.

**Discussion:**

Hyper-parameters tend to be sub-optimally fixed across different users rather than individually adjusted or even specifically set for a decoding task. The relevance of each parameter to the optimization problem and comparisons between algorithms can also be difficult to track with the evolution of the decoding problem. We believe that the proposed decoding pipeline and BO approach is a promising solution to such challenges surrounding hyper-parameter tuning and that the study’s findings can inform future design iterations of neural decoders for adaptive DBS and BCI.

## 1. Introduction

The successful adoption of deep brain stimulation (DBS) in the treatment of neurological disorders has prompted interest in subcortical brain regions as alternative invasive recording sites for brain-computer interface (BCI). Pathways through the basal ganglia and thalamus are heavily involved in the preparation and regularization of movement. These motor-control mechanisms are, in part, described by an asymmetry between anti-kinetic beta (12–32 Hz) and pro-kinetic gamma (32–90 Hz) waves measured in local field potentials (LFPs) (Cassidy et al., 2002; Brown, 2003; Anzak et al., 2012; Tan et al., 2013, 2016). These waves are useful biomarkers for neural decoding. Stimulation triggered during hyper-synchronous beta rhythms of LFPs in the subthalamic nucleus (STN) can help alleviate Parkinsonian rigidity and bradykinesia symptoms (Little et al., 2013). Since, other pathological and physiological states have been identified from subcortical LFPs, including tremor (Bakstein et al., 2012; Camara et al., 2015; Hirschmann et al., 2017; Tan et al., 2019; Yao et al., 2020; Zhu et al., 2020; He et al., 2021), dopamine medication effects (Sand et al., 2021), pain (Zhang et al., 2013; Luo et al., 2018), sleep stages (Chen et al., 2019), finger pressing (Mamun et al., 2015; Golshan et al., 2018, 2020; Niketeghad et al., 2018), reaching actions (Golshan et al., 2018, 2020), left- or right-hand selection (Mamun et al., 2015), speech (Golshan et al., 2018, 2020), behavioral tasks (Zaker et al., 2016; Golshan et al., 2018, 2020), isometric force (Shah et al., 2016, 2018; Tan et al., 2016), body orientation (Mace et al., 2014), and gait stages (Tan et al., 2018). Deep brain LFPs decoding supports adaptive DBS treatment (He et al., 2021) and could foster the development of new BCI applications, especially aimed at DBS patients. These can, for instance, aim at neurofeedback therapy through gamification (He et al., 2019) or drive wearable devices to assist patients with impaired mobility (Benabid et al., 2019).

This article investigates a hyper-parameter automated tuning approach for STN-LFPs decoders. A hyper-parameter is a parameter space that cannot be directly found through a fitting algorithm (the most common being regularization constants). For simplicity, these will be referred to simply as a parameter from thenceforward. For decoding based real-time BCI or adaptive DBS, the pipeline tends to be separated into several stages, such as extracting relevant features, selecting channels, classifying, and stage transition. The impact of individual parameters in different stages of the processing pipeline has typically been gauged independently from one another (Shah et al., 2018; Yao et al., 2020). As algorithms evolve toward practical applications, increased complexity almost always results in larger parameter spaces (Mace et al., 2014). Manual tuning can become tedious when assuming no prior knowledge of the relevance of each parameter. Therefore, parameters are often sub-optimally selected based on preliminary assessments and maintained constant across all testing participants. When assuming limited computational resources, grid searching becomes impractical in large search hyper-volumes (Bergstra and Bengio, 2012). Gradient-based optimization constrains the problem to the differentiable functions and is not well suited for small-size datasets (Martineau et al., 2020). Other methods, such as genetic algorithms, have also been proposed. However, this more complex optimization approach necessitates expert knowledge in selecting the necessary genetic operators and correctly interpreting the optimization process (Mace et al., 2014), which may discourage its adoption.

In this study, Bayesian optimization (BO) was initially chosen for its ease of implementation. It employs predictive Bayesian modeling to iteratively sample the parameter space of a “black-box” objective function (Shahriari et al., 2016). Unlike random search (RD), BO uses past samples to make the best-informed sampling decision and guarantees better outcomes within a limited sampling budget (Shahriari et al., 2016). In BCI research, BO has been principally dedicated to the tuning parameters related to the machine learning stages (Ahmadi et al., 2019; Nandy et al., 2019). However, Bashashati et al. (2016) have shown that BO can be used to fine-tune feature extraction and spatial filtering parameters. The foreseen advantages of BO are threefold. First, it could completely automate and unify the tuning process. Secondly, it could improve decoding performance over what is achieved with a set of known parameters (drawn from what is typically used in other past studies) fixed for different users. Thirdly, as a model-driven approach, BO intrinsically builds a representation of the tuning process mapping parameters to the optimization objective. Parameter importance can thus be determined, and the BO model can also be iteratively updated for sequential tuning (Grado et al., 2018). As pointed out by Grado et al. (2018), a BO algorithm would not be deployed alongside the decoder algorithm on a device firmware (e.g., a DBS micro-controller unit). Instead, it would operate remotely and intermittently suggest parameter changes to said device based on transmitted LFP data, thus providing a secondary outer and long-time-scale adjustment loop tuning.

Moreover, asynchronous decoding detects a state change based on temporal events present within a small analysis window extracted from a continuous data stream (Nicolas-Alonso and Gomez-Gil, 2012). Although it is required for many practical applications, such as adaptive DBS, asynchronous decoding from STN-LFPs remains challenging, especially for the detection of sustained actions in time (Mace et al., 2014; Zaker et al., 2016; Niketeghad et al., 2018; Martineau et al., 2020). Reasons include poor signal-to-noise ratio and non-stationary behaviors of the feature signals (Niketeghad et al., 2018; Yao et al., 2020), high focality of the signal source in space across the electrode contacts (Anzak et al., 2012; Tan et al., 2013; Zhang et al., 2018), and significant inter-subject and inter-trial variances (Tan et al., 2013; Niketeghad et al., 2018). Therefore, several parameters must be set for asynchronous decoding, including feature extraction window length, selected recording channels, or the number of past feature samples considered for classification. An action [isometrical power gripping actions, as defined in Martineau et al. (2020)] detection task was chosen in this study to assess the feasibility of STN-LFP asynchronous decoding further.

Finally, although the offline correlation of movement with LFPs can be significant, in most previous studies, those correlations are based on average across multiple trials. As emphasized in Mace et al. (2014), Niketeghad et al. (2018), and Shah et al. (2018), the restrictions imposed by a real-time system (those being limited computational resources and memory allocation, causal operation on the data) are intrinsically linked to those of asynchronous decoding and often omitted, notably in the feature extraction stage. Estimated performances made offline for investigative purposes or parameter tuning need to represent online performance. The last requirement was thus to ensure that the decoder could be deployed in real-time, i.e., be refreshed online using streaming data. Many time-frequency decomposition techniques have been proposed to extract information about neural oscillations (Nicolas-Alonso and Gomez-Gil, 2012). Each technique relies on different interpretations of the signal (e.g., signal partitioning in both the time and frequency domain) and hence on different parameterizations. The influence of these parameters could be assessed here using BO. In total, five different real-time extraction methods were tested and compared. Causality in the extraction stage and throughout the pipeline was maintained, i.e., every operation used the current input state or past states stored in memory.

## 2. Materials and methods

**FIGURE 1:**
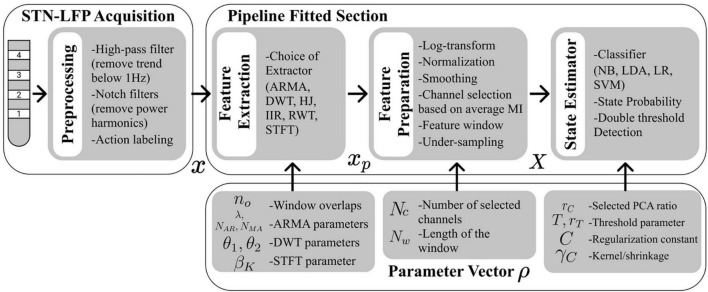
Flow diagram summarizing the sequence of processes for decoding and the different parameters assigned to them. Pre-processing was done prior to decoder pipeline fitting and tuning. A decoder pipeline had three main stages: a feature extraction (using either the ARMA, DWT, HJ, IIR, RWT, STFT method), a feature preparation stage including channel selection based on mutual information (MI) ranking, and a classifier (using either the NB, LR, LDA, SVM-PCA) stage including thresholding. Parameters in each of these three stages were tuned using BO.

**FIGURE 2:**
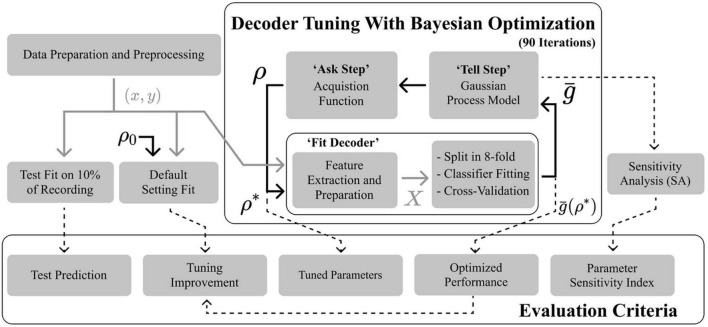
Flow diagram summarizing the Bayesian optimization and decoder fitting procedures, with associated evaluation criteria. Data is prepared in a pair (*x*,*y*) of neural signal and detection target and passed on to the optimizer. Ten percent of the recording is set aside for testing. The optimizer iteratively fits the pipeline presented in Figure 1 to yield a score $\bar{g}$. The first parameter vector ρ evaluated is the default vector ρ_0_. After what the optimizer fits a surrogate model mapping ρ to $\bar{g}$. An acquisition function uses the surrogate model to predict the next ρ. After a fixed number of iterations, the tuned parameters, optimal score, and improvement on default performance are recorded. Parameter importance is obtained through a sensitivity analysis of the surrogate model.

The overall decoder architecture is shown in Figure 1. The STN-LFPs signals were first recorded and pre-processed. Feature extraction was performed, followed by channel selection, feature preparation, and state estimation. Figure 2 presents the BO-based tuning process, and it repeatedly fitted and evaluated the decoder pipeline to select optimal parameters. The decoders and the BO procedure were implemented in python using the SciPy (Virtanen et al., 2019), NumPy (Harris et al., 2020), Scikit-learn (Pedregosa et al., 2011), Imbalanced-learn (Lemaître et al., 2017), and Scikit-optimization (Head et al., 2021) libraries.

### 2.1. DBS recording dataset

**FIGURE 3:**
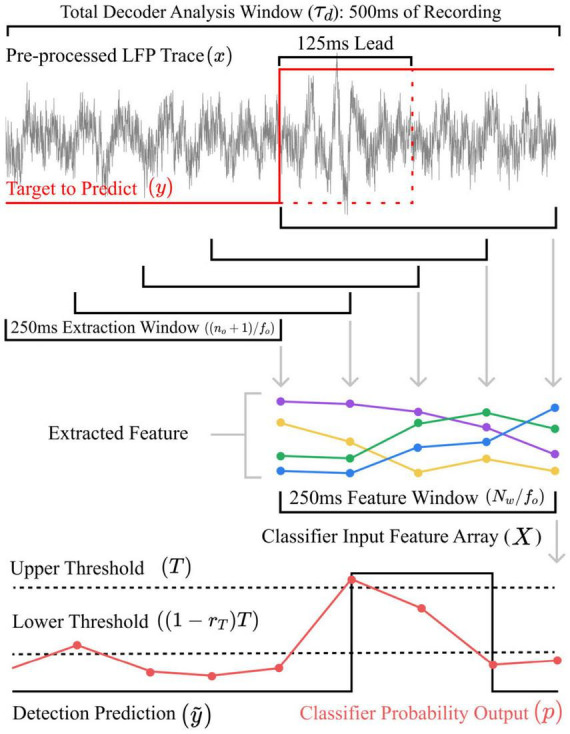
Flow diagram illustrating the feature extraction and detection processes in time, using, in this instance, 250 ms long extraction and feature epoch windows. The target label *y* is first shifted forward by a lead time of 125 ms. The classifier is thus trained to make prediction two update steps ahead. Features are extracted from *x* using a first moving window. They then accumulate in a feature vector *X* using a second moving window The classifier produces a probability signal *p*. The predicted state $\tilde{y}$ alters when *p* triggers the double threshold scheme.

Recordings collected from six test participants, previously reported in Tan et al. (2016) and Shah et al. (2018), were used in this study. Three participants were tested using their left and right hands, adding up to nine individuals’ datasets (Table 1). For practicality, recording across different hemispheres will be assumed to be independent, granted that factors, such as electrode type, physiology or medication may cause some dependencies. The participants were patients with Parkinson’s disease undergoing DBS surgery targeting the STN. Each gave informed consent to participate in the study, which was approved by the local ethics committee board. The participants were asked to apply a given amount of grip force for a given period following the presentation of a visual or auditory queue. Precise details on the data recording procedure can be found in Tan et al. (2013, 2016). Table 1 presents the number of trials and channels recorded. On average, a recording was 402 ± 105 s long and contained 40 ± 9 trials. Each grip was held for 4.1 ± 1.4 s. The ratio of grip against rest was 31 ± 7%, and the average holding force was 47 ± 27% of the maximum voluntary contraction. In each dataset, multiple channels of LFPs were recorded from lateral and contra-lateral electrodes (Table 1). The LFP signals were pre-processed following the same method outlined in Martineau et al. (2020). In summary, the prep-processing steps include detrending the recordings using a low-pass filter, removing main artifacts using notch filters, scaling the signals based on their standard deviation and resampling at the desired frequency. The dataset obtained was the following: an LFPs array *x* with time and electrode channel dimensions (for both hemispheres) such that *x* ∈ ℝ^*N*_*k*_×*N*_*ch*_^, where *N*_*k*_ is the total number of samples and *N*_*ch*_, the total number of channels. *x*was re-sampled at a rate of 512 Hz, which was defined as the decoder input sampling frequency *f*_*i*_. The decoder output $\tilde{y}$ matched the detection target *y* at a rate of 16 Hz, defined as the decoder output sampling frequency *f*_*o*_. Decoders typically operate in the range of 10–20 Hz (Shah et al., 2018; He et al., 2021). A base 2 number was chosen to facilitate downsampling, especially for implementing the discrete wavelet transform-based feature extractor. As shown in Figure 3, a sampling step-down *R* = *f*_*i*_/*f*_*o*_ was performed such that $\tilde{y}\,\left[k\right],y\,\left[k\right]\in \left\{0,1\right\}\,\forall k\in \left\{0,1,\ldots \,\frac{N_{k}}{R}-1\right\}$ with 0 marking a rest state and 1 an action state. A target lead of 125 ms was also introduced (Benabid et al., 2019). It is common practice to predict the target a few decoder update steps (in this case, two update steps) in advance to compensate for potential latencies introduced by processing, communication or even stimulator ramp-up (Tarvainen et al., 2004; Gruenwald et al., 2017; Benabid et al., 2019). Finally, 10% of the length of each recording was set aside for testing after tuning.

**TABLE 1:** Participant details, including DBS electrode channels available for recording and laterality.

Participant ID	PD01R	PD02L	PD02R	PD03L	PD04L	PD05L	PD05R	PD06L	PD06R	Mean ± SD
Contralateral channels	4	4	3	4	4	4	4	4	2	4
Ipsilateral channels	4	3	4	0	4	4	4	2	4	3
Number of trials	26	22	24	21	20	34	34	49	40	30 ± 9
Recording duration (s)	383	337	335	240	257	512	453	553	530	402 ± 105
Grip duration (mean ± SD, s)	2.95 ± 0.64	5.37 ± 0.19	5.28 ± 0.67	5.95 ± 1.54	3.25 ± 0.16	5.10 ± 1.33	3.61 ± 0.91	3.30 ± 1.72	3.79 ± 1.34	4.1 ± 1.4
Ratio of grip to rest (%)	20	35	33	49	25	34	27	29	30	31 ± 7
Grip level ratio (mean ± SD, %)	33 ± 19	0.82 ± 12	68 ± 15	69 ± 17	55 ± 28	37 ± 26	29 ± 21	39 ± 24	44 ± 28	47 ± 27

PD denominates Parkinson’s disease, L indicates that participant used their left hand to execute an isometric grip action. R indicates their right hand. A trial consists of a single isometric grip action including onset, holding, and relaxation stages. Grip duration is the time from the beginning of the action to its end averaged across trials. Grip ratio indicates the class balance of action vs. rest time points for every dataset. Grip level ratio is the trial peak force relative to the maximum force exerted in the recording averaged across trials.

### 2.2. Feature extraction

Five different frequency-domain and real-time extraction methods, including a bank of infinite impulse response (IIR) band-pass filters, a short-time Fourier transform (STFT), a recursive wavelet transform (RWT), a parameterized discrete wavelet transform (DWT), an adaptive auto-regressive moving average (ARMA) filter, based method, were implemented.

Filter banks have been proposed as a computationally efficient solution to extract standard electrophysiological sub-bands (Zhu et al., 2020). The method proposed by Gruenwald et al. (2017) was followed to construct a bank of fourth-order IIR Butterworth filters. The frequency band were defined as θ (1–8 Hz), α (8–12 Hz), β (12–32 Hz), γ_1_ (32–50 Hz), γ_2_ (50–100 Hz), γ_3_ (100–256 Hz), leading to a total of *N*_*b*_ frequency bands. The power of each sub-band $x_{p}\in {\mathbb{R}}^{\frac{N_{k}}{R}\times N_{c\,h}\times N_{b}}$ was estimated from the signal by measuring the variance of the sub-signals on a window length *L* and strides *R*. *L* was defined as a multiple of *R* such that:


(1) $$L=R\,\left(n_{o}+1\right)$$

where *n*_*o*_ is the window overlap number. A maximum *L* of 250 ms was allowed as in Flint et al. (2012), such that *n*_*o*_ ∈ ⟦0; 3⟧.

Secondly, the STFT is a reference time-frequency estimation method used in previous STN-LFP decoding studies (Bakstein et al., 2012; Hirschmann et al., 2017; Shah et al., 2018). The same epoching parameterization was used and the Kaiser window was chosen to parameterize the window shape through a single parameter β_*K*_ ∈ [0;15]. The STFT yielded a complete time-frequency transformation $\chi \in {\mathbb{C}}^{\frac{N_{k}}{R}\times N_{c\,h}\times \left(\frac{L}{2}+1\right)}$ of *x*. The same *N*_*b*_ sub-bands were extracted by averaging the correct frequency bins following the power operator χ[*k*,*l*,*n*]χ*[*k*,*l*,*n*] to obtain *x*_*p*_. A drawback of the STFT is that the resolution of the transform is directly dependent on *L*. With a small number of or no overlaps, narrower lower bands are thus not properly represented (in particular α) and not included as features.

This problem is typically addressed by opting for the continuous wavelet transform (CWT) instead. In addition to better capturing non-stationary signal behaviors, the CWT allows for multi-resolution signal analysis. The Morlet wavelet in particular is predominantly used in the field and thus is also regularly employed in decoder architectures (Bashashati et al., 2016; Tan et al., 2019; Golshan et al., 2020). To reduce the computational cost and latency of the CWT, Ren and Kezunovic (2010) proposed a complex sinusoid wavelet function, ψ, shaped with an asymmetric fifth-order polynomial envelope:


(2) $$\psi \,\left(t\right)=\left(-\frac{8}{9\,\sqrt{3}}\,{\left(\pi \,t\right)}^{3}-\frac{8}{27}\,{\left(\pi \,t\right)}^{4}-\frac{32}{135\,\sqrt{3}}\,{\left(\pi \,t\right)}^{5}\right)\,e^{2\,\pi \,\left(\frac{1}{\sqrt{3}}+j\right)\,t}$$

Ren and Kezunovic (2010) demonstrated that the CWT with ψ can be recursively expressed. This RWT approach was implemented as a complex IIR filter bank. Each filter projected the signal on a wavelet basis. Four wavelets were used per octave between 1 and 256 Hz, or a total of 32 filters, yielding a time-frequency transformation χ ∈ ℂ^*N*_*k*_×*N*_*ch*_×32^. The power array $x_{p}\in {\mathbb{R}}^{\frac{N_{k}}{R}\times N_{c\,h}\times N_{b}}$ was obtained after a power operation and application of epoch averages on χ.

Moreover, the more common alternative to the CWT is the DWT (Bakstein et al., 2012; Mamun et al., 2015; Luo et al., 2018). The DWT is implemented using small and efficient filter bank which samples discrete points in the CWT. The main design difficulty associated with the DWT is the choice of a mother wavelet ψ (Mamun et al., 2015; Luo et al., 2018). Farina et al. (2007) proposed using the parameterized lattice realization of the quadrature mirror filter to shape the mother wavelet to neural signals which in turn helped increase decoding performance. Each node in the DWT decomposition tree was constructed using a three-tap filter. The quadrature mirror filter split a signal *x* into low *x*_*g*_ and high *x*_*h*_ components through two rotations $\theta_{1},\theta_{2}\in ]\frac{\pi }{2};-\frac{\pi }{2}[$ (Vaidyanathan, 1993; Farina et al., 2007). To match *f*_*o*_, *N*_*l*_ = *log*_2_⁡(*R*) levels were required. The decomposition tree recursively filtered *x*_*g*_ at each level *l*, resulting in a set of wavelet coefficients $S_{c}=\left\{x_{h}^{1},x_{h}^{2},\ldots \,x_{h}^{l},\ldots \,x_{h}^{N_{l}},x_{g}^{N_{l}}\right\}$. The power array $x_{p}\in {\mathbb{R}}^{\frac{N_{k}}{R}\times N_{c\,h}\times \left(N_{l}+1\right)}$ was assembled by epoching each coefficient of *S*_*c*_ with a matching stride $R_{l}=\frac{R}{2^{l}}$, length *L*_*l*_, and computing the sum of squares.

Last, a parametric modeling and spectral estimation methods have been regularly used in STN-LFP (Foffani et al., 2005; Mamun et al., 2015; Shah et al., 2018) and other neural decoders (Chisci et al., 2010). The recursive-least-square ARMA scheme proposed by Tarvainen et al. (2004) was here investigated. The algorithm extracted the ARMA coefficients *x*_*c*_ ∈ ℝ^*N*_*k*_×*N*_*ch*_×(*N*_*AR*_ + *N*_*MA*_)^ from *x*. *N*_*AR*_ ∈ ⟦1, 12⟧,*N*_*MA*_ ∈ ⟦0, 6⟧ are the number of auto-regressive and moving average coefficients, respectively (Tarvainen et al., 2004). *x*_*c*_ was updated using a forgetting factor λ ∈]0,1[. Epoching served to average and downsample *x*_*c*_ before estimating of the power spectrum (Tarvainen et al., 2004). The spectrum was partitioned into *N*_*b*_ bands to obtain *x*_*p*_.

In addition, the usage of Hjorth (HJ) parameters (activity, mobility, and complexity) (Shah et al., 2018; Zhu et al., 2020; He et al., 2021) was investigated. The signal first, *x*_*d*_, and second derivatives were estimated using discrete differences. The parameters, *h*_*1*_, *h*_2_, and *h*_*3*_, were then computed by taking the following the signal variance on epochs of length *L* and stride *R*:


(3) $$h_{1}\,\left(x\right)={\text{Var}}_{\text{LR}}\,\left(x\right)$$

(4) $$h_{2}\,\left(x\right)=\sqrt{\frac{{\text{Var}}_{\text{LR}}\,\left(x\right)}{{\text{Var}}_{\text{LR}}\,\left(x_{d}\right)}}$$

(5) $$h_{3}\,\left(x\right)=\frac{h_{2}\,\left(x_{d}\right)}{h_{2}\,\left(x\right)}$$

where Var_LR_ is the epoch-variance operator. Hjorth parameters indirectly capture some characteristics of the signal spectrum through the time domain (Hjorth, 1970). Compared to most other time-frequency methods, HJ parameters are inexpensive to compute, making them desirable for real-time applications (Zhu et al., 2020; He et al., 2021).

A computational benchmark was conducted for every feature extraction method by measuring the time taken to extract features from a fix length (100 s) random signal and estimating the time taken to process a signal block of size *R*. Under real-time constraints, such a block is needed to update the decoder at a rate *f*_*o*_. The computational time, thus, needs to be inferior to 1/*f*_*o*_ (or 62.5 ms). The SciPy implementation (Virtanen et al., 2019) of the STFT algorithm was used partly to serve as a reference, partly because it is well-optimized and uses the fast-Fourier transform algorithm. Given the relatively large size of the extraction windows, a direct filter implementation would have been inefficient. Aside from the STFT, all extractors were implemented using the NumPy library (Harris et al., 2020). In every extractor, every filter was recursively updated at each time step to imitate real-time conditions. When implementing the step update, the well-optimized multi-dimensional array operation capabilities of NumPy (Harris et al., 2020) were carefully considered.

### 2.3. Channel selection and feature window

From a real-time deployment perspective, it could be costly to carry signal acquisition on all channels, extract all features and only select a sub-set for decoding. Instead, a channel selection approach was chosen whereby channels can be de-activated if non-selected. Channels were selected based on the cumulated mutual information (MI) of their extracted features in relation to the decoded target. Given *x*_*p*_ and *y*, the average information content $\bar{M\,I}$ of the *l^th^* channel was defined as:


(6) $$\bar{M\,I}\,\left[l\right]=\frac{1}{N_{i}}\,\sum_{i=0}^{N_{i}-1}M\,I\,\left(x_{p}\,\left[k,l,i\right],y\right)$$

where *MI* is the mutual information operator (Pedregosa et al., 2011; Ross, 2014), *N*_*i*_ is the total number of features for each channel, so either *N*_*b*_, *N*_*l*_ + 1 (if DWT is used) or 3 (if HJ is used), and *l* is the channel index. $\bar{M\,I}$ was sorted in descending order. *N*_*c*_ ∈ ⟦1,*N*_*ch*_⟧ top ranked channels were selected. Features of the selected channels were further processed by applying a logarithm transform, normalization, and further smoothing using the feature-wise Kalman filter proposed by Gruenwald et al. (2017) (except for the ARMA extractor, where it could be adjusted through λ). The feature matrix $X\in {\mathbb{R}}^{\frac{N_{k}}{R}\times N_{f}}$, with Nf=Nc⁢Nw⁢Ni, was assembled by flattening all the channels and bands (or coefficients in the DWT case, or parameters in the HJ case) dimensions and concatenating *N*_*w*_ ∈ ⟦0; 15⟧ past time points to form a moving window of features (or a maximum length of 1 s) (Flint et al., 2012; Shah et al., 2018; Tan et al., 2019; He et al., 2021). The feature and label pair (*X*,*y*) were under-sampled according to repeated-edited-nearest-neighbors method to create a more balanced training set before classifier fitting (Tomek, 1976; Lemaître et al., 2017).

Furthermore, the parameters *n*_*o*_, *N*_*c*_ and *N*_*w*_ controlled the total decoder window τ_*d*_ (Figure 3) and the total number of features *N*_*f*_. These two metrics were used to evaluate the complexity of the resulting decoder. A decoder with a smaller τ_*d*_ established more temporally located relationships between neural features and the decoded state. Practically, decoder with longer τ_*d*_ required more memory to store past signal epochs for extraction and past feature epochs for state estimation. Likewise, *N*_*f*_ also directly influenced memory storage and, more importantly, the computational cost of updating the state estimator. Tuning *N*_*c*_ and *N*_*w*_ also acted as a form of feature selection, which could help reduce model complexity.

### 2.4. State estimator and objective function for detection

Four different classifiers retained from He et al. (2021) were tested to estimate *y* from *X*: (1) the Gaussian Naïve Bayes (NB) classifier was chosen for its lack of tuning parameters (Mamun et al., 2015). The NB served as a special case to evaluated if decoder parameters (e.g., *n*_*o*_, *N*_*c*_, or *N*_*w*_) other than fitting regularization constants could be leveraged to increase performance. (2) The linear-discriminant analysis (LDA) classifier has been shown to deliver good decoding performance (Flint et al., 2012; Gruenwald et al., 2019). A shrinkage parameter γ_*c*_ ∈ [0,1] was used to regularize the covariance estimation of *X*. (3) Logistic regression (LR) has successfully been applied to STN-LFPs decoding (Shah et al., 2016; Tan et al., 2019; He et al., 2021), particularly for sparse model fitting using*L*_*1*_-norm regularization. The regularization was set through a parameter *C* ∈]0,5]. Its setting acted in part as a form of feature selector. (4) Thanks to their versatile non-linear kernels, support vector machine (SVM) classifiers have been regularly employed for high-performance decoding (Mamun et al., 2015; Golshan et al., 2016, 2018; Nandy et al., 2019; He et al., 2021). The radial-basis variant was chosen. It required a regulation and kernel parameter *C*,γ_*c*_ ∈]0,5] (Nandy et al., 2019). Given the expandable nature of the feature space (of size *N*_*F*_), the computational demand of the SVM fitting algorithm and the fact that decoder fitting occurred across full-length time-series at each BO iteration, principal component analysis (PCA) dimensionality reduction had to be introduced to compress *X* by a factor *r*_*c*_ ∈]0,1] (Golshan et al., 2018). The maximum number of selected components was as well capped at 64. Once deployed, the computational cost of operating the NB, LDA, and LR scales with *N*_*f*_. For the SVM, it also scales with the number of support vectors used by the classifier. Other non-linear methods such as simple decision trees and nearest-neighbor algorithm were not considered as they were found to be less performant (He et al., 2021). In all, a range of incrementally more sophisticated, and arguably more complicated to tune, classifiers was chosen.

Moreover, to denoise the output prediction of the classifier, a pair of thresholds operated as a transition trigger. Given the classifier probability *p*(*y* = 1)[*k*], the predicted state $\tilde{y}\,\left[k\right]$ was given by:


(7) $$\tilde{y}\,\left[k\right]=\left\{ \begin{array}{ccc} \begin{array}{c} 1\,\text{if}\\ 0\,\text{if} \end{array} & \begin{array}{c} p\,\left[k\right]>T\\ p\,\left[k\right]<\left(1-r_{T}\right)\,T \end{array} & \begin{array}{c} \text{and}\,\tilde{y}\,\left[k-1\right]=0\\ \text{and}\,\tilde{y}\,\left[k-1\right]=1 \end{array} \end{array}\right.$$

where *T* ∈ [0,1] is the upper threshold, and *r*_*T*_ ∈ [0,1] is the position of the lower threshold relative to *T*. Thresholds are typically set at the optimal point of the receiver operating characteristic (ROC) curves, balancing the sensitivity and specificity of a classifier. Unfortunately, this curve cannot be drawn for a dual-threshold system. Instead, the geometric mean *g* between sensitivity and specificity was chosen to estimate an optimal trade-off (Cai et al., 2010). Given the detection true-positive rate (TPR) and false-positive rate (FPR), *g* was given by:


(8) $$g=\sqrt{T\,P\,R\,\left(y,\tilde{y}\right)\times \left(1-F\,P\,R\,\left(y,\tilde{y}\right)\right)}$$

If *TPR* = 0, then *g* = 0. Likewise, if *FPR* = 1, then *g* = 0. Consequently, *g* only leans toward *1* if both objectives are balanced, thus avoiding the extreme situation where, for instance, the state-estimator only detects true positives or negatives. On a traditional ROC diagram, *g* would indicate a curve point sticking furthest toward TPR = 1 and *FPR* = 0. An eightfold cross-validation was employed to evaluate the average detection performance $\bar{g}$. The number of folds was chosen to be double that of the number of cores available on the processor (Intel^®^ Core i7-7700K 4.20 GHz) to accelerate the evaluation of the folds through parallelization.

### 2.5. Decoder parameter tuning

Figure 1 illustrates different parameters introduced in the different processing stages and, Figure 2 the BO procedure. BO was implemented to optimize $\bar{g}$ using the scikit-optimize library (Head et al., 2021). The parameters formed a bounded search space *S* from which a parameter vector ρ ∈ *S* was sampled. To quantify the performance increase resulting from tuning, initial quantities, reflecting values typically found in other studies, formed a default parameter vector ρ_0_. A 3-tap Daubechies wavelet was used for the DWT by default (Rieder et al., 1998). A summary of all tuned parameter, search intervals and default values is given in Table 2. Given $\bar{g}$ as a function of *x*, *y*, and ρ, the best parameters ρ*were defined as:


(9) $$\rho^{*}=\arg\,\max_{\rho \in S}\bar{g}\,\left(\rho ,x,y\right)$$

**TABLE 2:** Summary of search space *S* and default vector ρ_0_.

Pipeline	*n*_*o*_	*N*_*c*_	*N*_*w*_	*T*	*r*_*t*_
*S*	⟦0; 3⟧	⟦1, *N*_*ch*_ ⟧	⟦0; 15⟧	[0, 1]	[0, 1]
ρ_*o*_	3	2	0	0.5	0
**Extractor**	**STFT**	**DWT**	**ARMA**		
	**β_*k*_**	**θ_1_,θ_2_**	**λ**	***N*_*AR*_**	***N*_*MA*_**
*S*	[0;15]	$]\frac{\pi }{2};-\frac{\pi }{2}[$	]0,1[	⟦1, 12⟧	⟦0, 6⟧
ρ_*o*_	5	(0.50,−0.105)	0.99	6	2
**Classifier**	**LR**	**LDA**	**SVM**		
	***C***	**γ_*c*_**	***r*_*c*_**	***C***	**γ_*c*_**
*S*	]0,5]	[0,1]	]0,1].	]0,5]	]0,1]
ρ_*o*_	1	0	0.1	1	0.1

n_o_ is the number of overlaps between feature extraction windows (with a maximum window of 250 ms). N_c_ is the number of channels selected amongst N_ch_ after ranking based on MI. N_w_ controls the length of the feature window (for a maximum of 1 s). T is the upper threshold placed on the output of the classifier. r_t_ controls the placement of the lower-threshold relative to T. β_k_ is the shape factor of the STFT window function. θ_1_,θ_2_ are the lattice parameters for the DWT. λ is the decay rate for the ARMA filter. N_AR_,N_MA_ control the coefficient settings for the ARMA filter. C are regularization constants for the LR and SVM. γ_c_ is respectively the shrinkage coefficient for the LDA and a kernel parameter for the SVM. r_C_ is the PCA ratio for the SVM.

The corresponding maximum cross-validation performance was noted as ${\bar{g}}^{*}\,\left(\rho^{*},x,y\right)$. The BO scheme repeatedly fitted and evaluated the pipeline at each iteration (until 90 iterations were reached). The algorithm was seeded by first evaluating ρ_*o*_. In a “tell” step, the optimizer fitted a surrogate model which mapped *S* to $\bar{g}$. In an “ask” step, the optimizer, following the policy of a given acquisition function, suggested a new ρ (Shahriari et al., 2016). A Gaussian-process with Matern Kernel (Bashashati et al., 2016) was chosen for the surrogate model alongside the Gaussian-process hedge acquisition function (Shahriari et al., 2016; Head et al., 2021).

Before optimizing every feature extractor and classifier pair, a preliminary comparison between BO and RD was conducted by optimizing the STFT-LR decoder for three participant recordings (PD01R, PD05R, and PD06L), which represent well different optimized performance levels within the population sample. The optimization procedure was, in this case, repeated five times with different random seeds to produce average convergence trends. Individual trends were obtained by noting for every *i*-th iteration of the maximum performance obtained on or before said iteration ${\bar{g}}_{i}^{*}$. Average cumulated regret trends were also produced for both algorithms (Shahriari et al., 2016). A regret trend *r*_*i*_ was defined as:


(10) $$\left\{ \begin{array}{c} r_{i}=r_{i-1}+\left({\bar{g}}_{i}^{*}-{\bar{g}}_{i}\right)\,\forall i=1,\ldots ,N_{i}\\ r_{o}=0 \end{array}\right.$$

where ${\bar{g}}_{i}$ is the score obtained on the *i*-th iteration and *N*_*i*_, the total number of iterations. An initial 60 iterations were set. ${\bar{g}}_{i}^{*}-{\bar{g}}_{i}$ measures the quality of every decision the optimizer takes. Perfect decision-making would find a new optimum point at every step such that ${\bar{g}}_{i}^{*}-{\bar{g}}_{i}=0$. Both convergence and regret trends gave insight into the progression of the optimization sequence.

### 2.6. Decoder evaluation and statistical analysis

Following the preliminary comparison, the gathered evaluation criteria summarized in Figure 2 were collected for every feature-extractor-classifier pair. The maximum cross-validation performance ${\bar{g}}^{*}$, *TPR* and *FPR* and the optimal parameter vector ρ* were recorded. The significance of the absolute improvement ${\bar{g}}^{*}-\bar{g}\,\left(\rho_{0},x,y\right)$ was evaluated using a Wilcoxon paired test. A Mood test was also performed between ${\bar{g}}^{*}$ and $\bar{g}\,\left(\rho_{0},x,y\right)$ to measure any reduction in inter-subject variance. Performance evaluation also included plotting the decoder output prediction on a test segment. Sensitivity analysis of the BO models was used to relate the contribution of each parameter to ${\bar{g}}^{*}$ using the SALib library (Herman and Usher, 2017). *S* was sampled using the Saltelli method (Saltelli, 2002), and the BO surrogate model was analyzed using the Sobol method (Sobol, 2001). The Sobol indexes measured the ratio of individual parameter input variances to surrogate model output variance (Sobol, 2001). Total order indexes for every parameter were recorded to capture both first-order and higher-order interactions (Herman and Usher, 2017). Analysis of variance (ANOVA) and *post-hoc* Tukey pairwise tests were applied to measure the dependence of $\bar{g}\,\left(\rho^{*},x,y\right)$, *TPR*, *FPR*, improvement, ρ*, τ_*d*_, *N*_*f*_ and parameter sensitivity indexes on the choice of extractor and classifier. Statistical testing was conducted using the SciPy (Virtanen et al., 2019) and Statsmodel (Seabold and Perktold, 2010) toolboxes. Figures were produced using the Seaborn (Waskom, 2021) and Matplotlib (Hunter, 2007) toolboxes.

## 3. Results

### 3.1. Preliminary comparison between random search and Bayesian optimization

**FIGURE 4:**
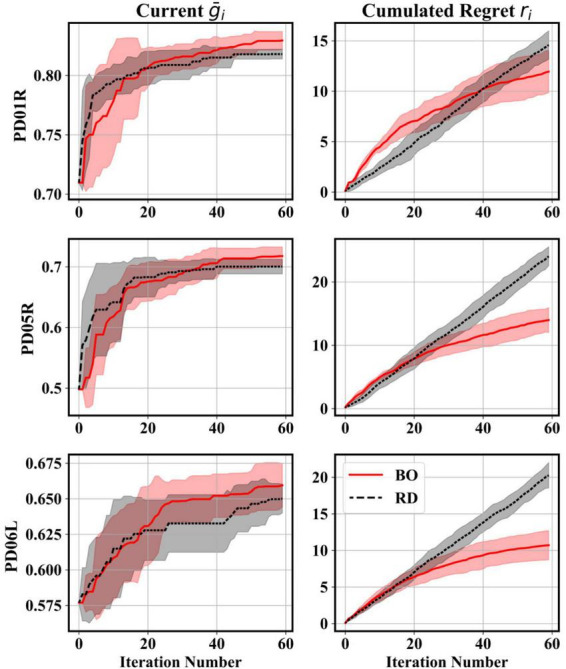
Comparison between BO and RD searches for three different participant recordings during the tuning of a STFT-LR decoder. The lines indicate the average convergence and cumulative regret trends. The shaded areas indicate 1 SD above and below the average. The average and SD at each iteration were taken by repeating the search 5 times. Convergence trends suggested that the RD made good initial guesses but was eventually overtaken by the BO. Regret trends showed that BO could make informed decisions compared to the RD.

Figure 4 presents averaged convergence and cumulated regret trends computed for three representative participant recordings. The trends indicated that the RD scheme was very effective at uncovering good initial parameter vectors, whilst BO had a rather slower convergence rate starting from ρ_*o*_. Nonetheless, after a few iterations, the convergence rate and plateau of the BO algorithm overtook that of the RD scheme. The cumulated regret trends confirmed that BO mapped sufficiently well the parameter space with each iteration, thus decreasing the ${\bar{g}}_{i}^{*}-{\bar{g}}_{i}$ through informed decision-making. As expected, since RD is memoryless, that gap remained constant, leading to a linear cumulation of decision errors.

Thus, for the presented decoder tuning problem, BO was better at ordering good solutions (i.e., ${\bar{g}}_{i}$ is more likely to be superior to ${\bar{g}}_{i-1}$, which is important for sequential learning) and at fine-tuning in later iterations. Consequently, for the remainder of the tuning runs, to bootstrap the BO search starting at ρ_*o*_, five additional random samples were drawn to seed the initial fit of the BO surrogate model. Thirty more iterations (adding to a total of *N*_*i*_ = 90 iterations) were added to allow for further fine-tuning.

### 3.2. Decoding performance

**FIGURE 5:**
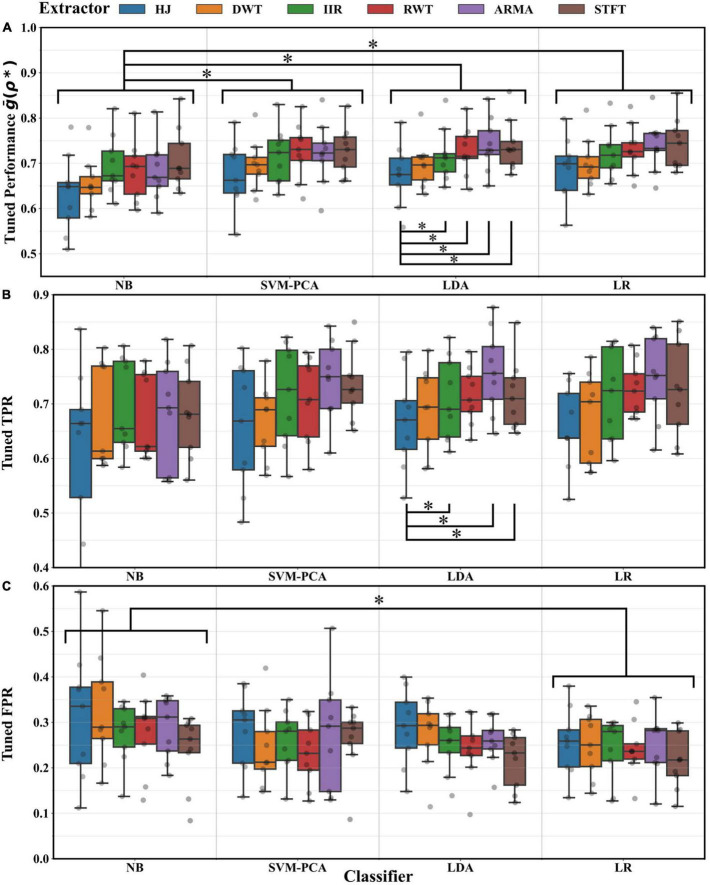
Maximal performance distribution across distributions and participants resulting from cross-validation averages after BO tuning for every extractor and classifier combination. **(A)** For the TPR and FPR geometric mean $\bar{g}$. Significant differences (*, Tukey test, *p* < 0.05) were found between decoders using the NB and those using other classifiers. Significant differences were also found between decoders using the HJ and those using other extractors. **(B)** For the TPR. Significant differences (*, Tukey test, *p* < 0.05) were found between decoders using the HJ and those using other extractors. **(C)** For the FPR. A significant difference was found between decoders using the NB and those using the LR classifier (*, Tukey test, *p* < 0.05).

Figure 5 reports the optimal cross-fold average $\bar{g}\,\left(\rho^{*},x,y\right)$, TPR and FPR achieved after BO tuning for every extractor and classifier pair. Medians laid within 0.6–0.75 for the $\bar{g}$ and TPR, and within 0.20–0.35 for the FPR. Combinations of ARMA STFT extractors with LDA and LR classifiers achieved the highest average $\bar{g}$ performance of 0.74 ± 0.06 (mean ± SD). ANOVA testing on $\bar{g}$ suggested that the choice of classifier and extractor impacted performance (*p* < 0.01). *Post-hoc* testing showed that all classifiers and extractors performed comparatively well except for the NB (*p* < 0.05) and the HJ (*p* < 0.05). The TPR depended only on the selected extractor (ANOVA, *p* < 0.01), with the HJ underperforming relative to the ARMA, IIR, and STFT (*post-hoc* tests, *p* < 0.05). Conversely, the FPR depended on the choice of classifier (ANOVA, *p* < 0.05), with the NB underperforming relative to the LR (*post-hoc* tests, *p* < 0.05).

**FIGURE 6:**
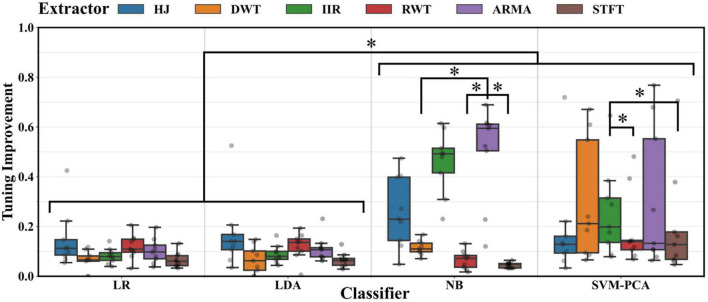
Distribution of performance improvement across participants when using BO tuned parameters over default parameters for every extractor and classifier combination. Significant differences (*, Tukey test, *p* < 0.05) were found between decoders using the NB, SVM-PCA on the one hand and those using the LDA, LR classifier on the other. Significant differences (*, Tukey test, *p* < 0.05) were found between decoders using the ARMA or IIR and those using other extractors.

Figure 6 reports the improvement in the overall decoding performance with the BO-based parameter optimization compared to that with default often used parameters fixed across all participants [$\bar{g}\,\left(\rho^{*},x,y\right)-\bar{g}\,\left(\rho_{0},x,y\right)$]. Paired testing showed that this gap was significant in every case (Wilcoxon, *p* < 0.05). ANOVA testing showed that the improvement gap depended again on the classifier and extractor pair (*p* < 0.01). *Post-hoc* testing indicated that improvement margins were tighter for the LR and LDA classifiers (*p* < 0.05). Default parameter solutions for the SVM-PCA and NB led to poorer decoding solutions. Lastly, no significant reduction in inter-subject variance was measured between default and optimized parameter performance (Mood, *p* > 0.05).

**FIGURE 7:**
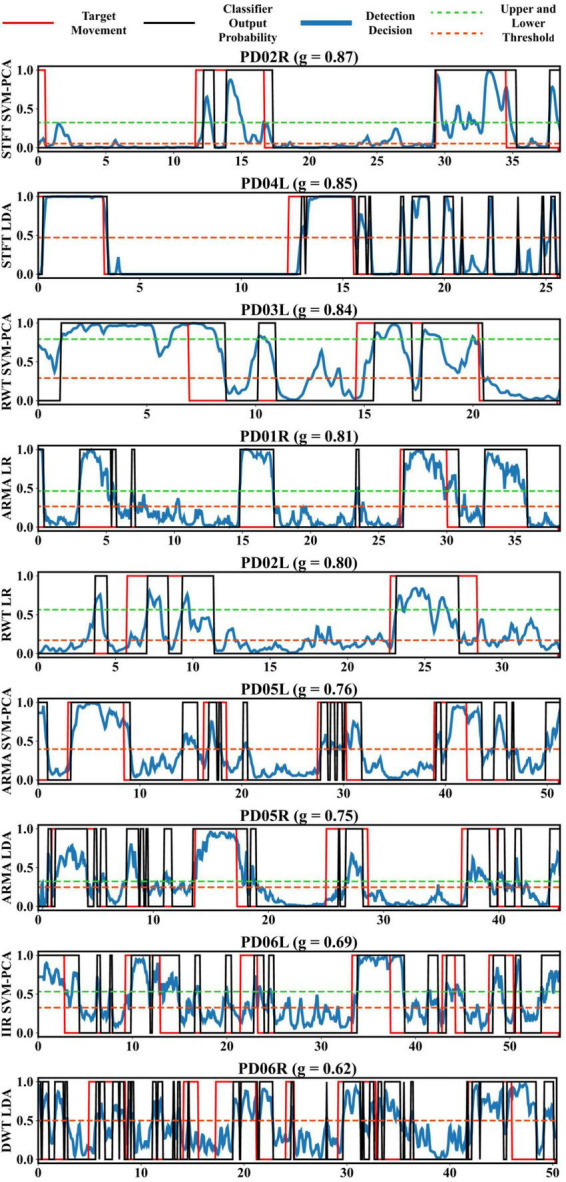
Best decoded test segment (10% of each recording length) for every participant with associated test score and extractor, classifier pair. Thresholds were set in an attempt to optimize detection rate based on the dynamics (range, latency, noisiness, or sudden changes) of the decoder output probability, where possible larger deadband helped avoid spurious transitions. The decoder had the option to remove the deadband by setting *r*_*T*_ to 0.

Figure 7 presents the best-decoded test performance for every user. The classifier output signal *p*[*k*] varied noticeably in terms of quality and temporal dynamics throughout the cohort. Effective detection occurred with high and sufficiently sustained *p* throughout the action with manageable levels of baseline noise (when *y*[*k*]=0) and action noise (when *y*[*k*]=1), such as observed in PD03L, PD04L, PD02L, and PD01R. In other instances, *p* adopted a more impulsive behavior, such as in PD02R or PD02L, making a full capture of the action difficult. Although the upper threshold prevented high baseline noise from triggering the detection, such as in PD03L, PD04L, or PD01R, it could not contend with very large spurious impulses outside of the action (when *y*[*k*]=0) as those found in PD04L, PD05R, PD06R, and PD06L. Conversely, spurious drops would also occur within the action (when *y*[*k*]=1) such as in PD05L, triggering the lower threshold.

### 3.3. Decoder parameterization

**FIGURE 8:**
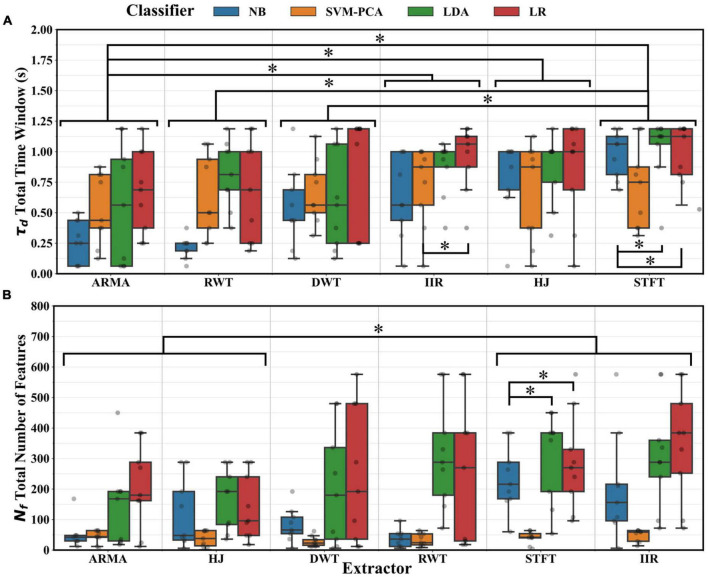
**(A)** Distributions across participants of the total decoder analysis windowτ_*d*_ using optimal parameters. Significant reductions (*, Tukey test, *p* < 0.05) were found between decoders using the ARMA, RWT, or DWT extractors and those using the IIR, HJ, or the STFT extractors. Significant differences (*, Tukey test, *p* < 0.05) were also found between decoders using the NB or LR and those using other classifiers. **(B)** Distributions of the total number of features *N*_*f*_ used for decoding. Significant reduction (*, Tukey test, *p* < 0.05) were found between decoders using the ARMA or HJ and those using the STFT or IIR extractors. Setting aside the SVM-PCA, significant reduction (*, Tukey test, *p* < 0.05) were also found between decoders using the NB and those using the LDA or LR classifiers.

Optimal parameter distributions were tested using ANOVA. The test results suggested that the thresholds (*T* and r_*T*_) did not depend on either the classifier or the extractor (*p* > 0.01) but rather on the intrinsic signal properties of individual recordings. The number of channels (*N*_*c*_) depended on the choice of classifier (ANOVA, *p* < 0.01) but not of the extractor. *Post-hoc* testing suggested that fewer channels were selected using the NB classifier rather than the LR or LDA (*p* < 0.05). The number of overlaps between windows (*n*_*o*_) was dependent on both estimator and extractor choices (ANOVA, *p* < 0.01). The STFT adopted a significantly larger extraction window length (*L*) than the ARMA, IIR, and HJ extractors; the LR, than the LDA and NB classifiers (*post-hoc* tests, *p* < 0.05). Likewise, the choice of feature window length (*N*_*w*_) depended on the extractor (ANOVA, *p* < 0.01) but not on the classifier selection. Notably, the STFT, IIR, and HJ required significantly larger *N*_*w*_ than the ARMA extractor (*post-hoc* tests, *p* < 0.05). Figure 8 presents distributions for the decoder analysis window (τ_*d*_) and the total number of features employed for decoding (*N*_*F*_, section 2.3). τ_*d*_ and *N*_*f*_ depended on the choice of extractor and classifier (ANOVA, *p* < 0.01). Because of the smaller optimum *N*_*w*_ and *n*_*o*_, the ARMA extractor and the NB classifier required significantly smaller windows than the STFT extractor and LR classifier (*post-hoc* tests, *p* < 0.05). Likewise, because of smaller *N*_*w*_, *N*_*c*_ or *N*_*i*_, the decoders using the ARMA and HJ extractors or the NB classifier required significantly fewer features than the STFT extractor or LR classifier (*post-hoc* tests, *p* < 0.05). Lastly, there was no dependence between optimal extractor parameters and classifier choice and no dependence between classifier parameters and extractor choice.

**FIGURE 9:**
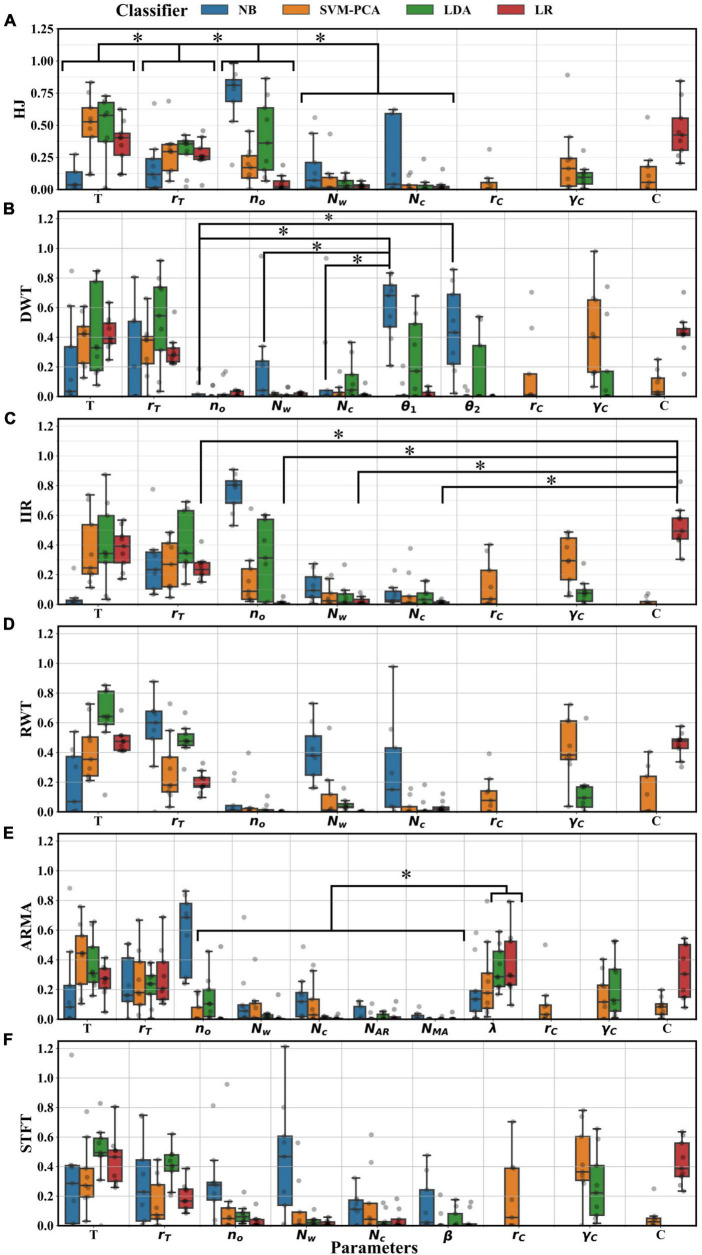
Distributions of sensitivity index for every tuned parameter across participants. Index resulted from a Sobol sensitivity analysis of each BO surrogate model. **(A)** Index distribution for decoders using the HJ; **(B)** DWT; **(C)** IIR; **(D)** RWT; **(E)** ARMA; and **(F)** STFT extractors. Significant decoder reduction (*, Tukey test, *p* < 0.05) in importance were found between *T*,*r*_*T*_,*n*_*o*_ and lastly *N*_*c*_,*N*_*w*_. In most extractor pairing, significant differences (*, Tukey test, *p* < 0.05) were found between most parameters and the regularization constant *C* of the LR classifier (showed in **C**). Significant differences (*, Tukey test, *p* < 0.05) were also found between the importance of λ when using the LR and LDA classifier and other parameter **(B)**. When paired with the NB, significant differences (*, Tukey test, *p* < 0.05) were found between lattice parameter θ_1_,θ_2_, and other parameters **(E)**.

The index distributions yielded from the sensitivity analysis are presented in Figure 9 for each parameter. Considering the subset of common parameters (*n*_*o*_,*N*_*c*_,*N*_*w*_,*r*_*T*_,*T*), ANOVA testing suggested that the importance indexes varied depending on the parameter type and the classifier selected (*p* < 0.01), but not on the extractor (*p* > 0.05). *Post-hoc* testing showed that the upper and lower thresholds in the final decoding stage (*T* and *r*_*T*_) were significantly more important than all other parameters (*p* < 0.05). The window overlap (*n*_*o*_) was significantly more important than the number of channels selected (*N*_*c*_) and the window length (*N*_*w*_) (*p* < 0.05), despite clear preferential settings depending on the selected classifier or extractor. *Post-hoc* tests also showed that the NB and the LDA attributed more importance to those parameters than the LR classifier (*p* < 0.05). *Post-hoc* tests were performed for extractor combinations with the LR classifier with all available parameters. Aside from *T*,*r*_*T*_, the regularization constant, *C*, was the most important parameter (*p* < 0.05), with the unique exception of the ARMA λ parameter. In contrast, with the NB classifier, all parameters had shared similar levels of importance (*p* > 0.05), except for *n*_*o*_, which was more important with ARMA and IIR pairing (*p* < 0.05). The remaining parameters had comparable importance with the LDA and SVM-PCA classifiers. Regularization parameters did not hold as much importance as they did with LR classifiers. Lastly, extractor-specific parameters other than λ (the model order, *N*_*AR*_,*N*_*MA*_, for ARMA, the lattice parameters, θ_1_,θ_2_ for DWT and the window shape factor, β_*K*_, for the STFT) were never significantly more important than other parameters (omitted some exceptions, Figure 9).

### 3.4. Computational benchmark for feature extraction

**FIGURE 10:**
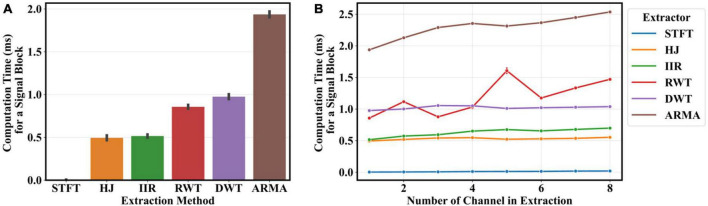
Computational time benchmark for every feature extraction method (using an Intel^®^ Core™ i7-7700K 4.20 GHz); each method was evaluated using a 100 s long, 512 Hz (*f*_*i*_), Gaussian random noise signal. **(A)** The extraction was repeated 30 times using default setting parameters and a single channel signal. The average time taken to complete a single computational step (process a block of *R* = 32 signal points) at 16 Hz (*f*_*o*_) was estimated. **(B)** The benchmark was reproduced by incrementally adding channels to a total of 8. Each method benchmark is significantly inferior to the refreshing time (62.5 ms) between decoder output updates. All methods were custom implemented using the NumPy library, except the STFT which used a specialized function from the SciPy library. Error-bars indicate the SD taken across the 30 extractions.

Figure 10 presents a benchmark to evaluate the computational time taken to process features for a single decoder step for each method. All of them were sufficiently fast to operate in real-time. A large overhead was observed when features were extracted from a single channel, most likely associated with slow array manipulation in python at each time-step. More complex methods such as ARMA and DWT were more penalized than others (more linear transformation for the ARMA and multi-level processing for the DWT extractor). The DWT should be, in practice, far more efficient (Farina et al., 2007). Nonetheless, extraction processing time was found to scale favorably as multi-dimensional signals were introduced. More demanding processes, such as the RWT, ARMA and, to an extent, the IIR extractors, had a notable increase in computational time with every added channel. Other methods, such as the DWT and HJ were more computationally efficient. The benchmark proposed in this study is specific to the programming language, operating system and processor. Different hardware and software optimization considerations should ultimately be made based on the target deployment device and the selected method.

## 4. Discussion

This investigation demonstrates the benefits of automatic parameter tuning using BO for STN-LFPs asynchronous decoding. The parameterization of a relatively complex decoding pipeline optimized a task-relevant objective function, and BO significantly increased task performance from default parameter settings and substituted part of the expert knowledge required for calibration. In addition, BO allowed us to systematically evaluate and compare different combinations of feature extraction and classification methods for decoding. It also helped to uncover the parameters that had the most impact on the decoding performance, such as the classifier thresholds and the window overlaps. In particular, the classifier thresholds were configured differently depending on the individual properties of the STN-LFPs signals.

### 4.1. Performance comparison and study limitations

The optimum performance obtained in this study is comparable to the results of past studies that have employed similar asynchronous and real-time constraints to decode STN-LFPs (Mace et al., 2014; Niketeghad et al., 2018). The limitation remains that stable detection can vary significantly across participants. We note, however, that BO did not reduce inter-subject variance, indicating that lower-performing cases may be due to a lack of information related to the decoding target in the recorded signals. Moreover, the performance increase can be achieved from the simultaneous optimization of the extractor and classifier, indicating that default parameters may not capture a priori expertise knowledge. A clear benefit of this approach was observed principally in the NB classifier. Nonetheless, end-to-end decoder optimization may not have been the most efficient approach in the proposed architecture and decoding task context. The tuning problem could potentially be broken down into several stages, e.g., one for feature extraction (optimizing some desirable attributes of *X*) and another for state estimation (optimizing $\bar{g}$). Separating these two stages would also be more practical to implement estimators with more computational intensive fitting such as the kernel SVM (and avoid resorting to PCA to compress *X*).

Furthermore, the decoding of movement in this investigation provides a basis to apply the method to other DBS targets, state types (e.g., tremor, gait, etc.), and BCI tasks to further gauge the impact of tuning. Online testing is needed to perform decoding sequentially with stimulation in an adaptive configuration (He et al., 2021), continuous tuning (Grado et al., 2018), and for deployment, accounting for the fixed computational and memory budget of an embedded device (Zhu et al., 2020). Finally, findings in this study can further guide algorithm selection, and a BO approach could help ease deployment by rethinking the task objective with these budgeting constraints in mind.

### 4.2. Improving the tuning method

The optimization process involved a range of parameterization trade-offs. For instance, channel ranking or other decomposition strategies are not a strict guarantee of improved performance through a reduction in classifier overfitting (Shah et al., 2018). Except for the NB, the BO algorithm kept adding channels despite diminishing returns in performance gain (Flint et al., 2012). A multi-objective approach or optimization constraints could narrow the set of reasonable tuning solutions. Penalty for delayed detection, output chattering, or model complexity could be introduced (Mace et al., 2014). Parameters such as the number of channels selected (*N*_*c*_) and the window length (*N*_*w*_) could then become more relevant. Further practical improvements could also be made to the BO algorithm implementation by introducing robust convergence criteria (Bashashati et al., 2016) and more adaptive acquisition functions (Grado et al., 2018).

### 4.3. Tuning and usage of the proposed feature extractors

For feature extraction, parameters influencing the temporal smoothness of the feature signals, such as the number of overlaps between feature extraction windows *n*_*o*_ and the ARMA forgetting factor λ were important to decoding. A second-order Kalman filter (Chisci et al., 2010; Yao et al., 2020) could be tuned using BO and applied either in the feature space for further smoothing or at the classifier output to help stabilize the state transition. Reducing the decoder output frequency *f*_*o*_ could also help reduce higher-frequency noise at the cost of a slower refreshing rate. An intermediate cost-function measuring signal-to-noise ratio could be employed to tune extractor parameters in the first optimization stage. Feature extractors overall delivered comparable levels of performance. With hindsight, testing other signal features that were not purely spectral-based (Bakstein et al., 2012; Wang et al., 2018) or different combinations of feature bands might have been more intriguing (Tan et al., 2016). The BO algorithm could have been used to select the combination of features better suited to decoding.

Although the extractor was not critically associated with the detection performance, this work suggests that the choice of extractor still influences extraction and feature window lengths and the number of features used for classification, elements important to the decoder design. Some extractors may thus be better suited to some decoding tasks than others. For instance, the ARMA extractor was among the more performant and instantaneous methods. It can also be helpful if connectivity features (Mamun et al., 2015) or instantaneous phase estimation (Foffani et al., 2005) are of interest. However, it comes at the cost of a specialized implementation and a more computationally intensive process. The STFT, in comparison, is a practical, efficient and versatile extractor. Since the window shape only had a limited influence on decoding, using, for instance, a recursive formulation of the exponential window (Grado et al., 2017) could help minimize windowing lag. Although, the performance with the RWT did not supersede that of the STFT, it has the benefit of combining a narrow-band filter in a compact IIR form with a complex phase estimator, which could be useful to isolate a single or few specific sub-band phase and power. Otherwise, the additional computational burden of a large number of relatively long complex filters associated with a full-signal decomposition does not seem justify. Smaller filter banks, such as the IIR extractor, are relevant to embedded applications (Zhu et al., 2020). The mutual information-based channel selection scheme could be extended to incorporate only informative power bands. Selected individual features across contacts is here more practical as only the relevant filters can be activated on a given channel greatly reducing the computational burden. Given the comparatively short τ_*d*_ for the DWT and its computational efficiency, BO could permit the optimization of longer wavelets which could help match the performance of other extractors. The packet transform could introduce a sparser and more adaptive signal representation (Mamun et al., 2015; Luo et al., 2018). Lastly, the HJ parameters were not sufficiently informative and should only be used as complementary features for movement decoding (Shah et al., 2018).

### 4.4. Tuning and selecting state estimators for decoding

The double-thresholds system played an important role in providing stable state transitions. It was observed that the upper threshold was generally set above spurious events to prevent false-positive triggering. A higher triggering level delayed detection, especially if the transient response of the feature extractor and classifier combination was slow. A lower second threshold, close to the baseline activity of the classifier output, was preferable. It aligned the detection and the action ending better and prevented a spurious transition if the classifier output suddenly dipped during an action. BO could thus help calibrate more complex state machines required for adaptive DBS (He et al., 2021).

Aside from the NB classifier, there was no substantial difference between the classifier types in terms of performance. However, not every classifier was best suited for the proposed detection task. Fitting the SVM-PCA classifier was more computationally demanding, given the temporal longitudinal aspect of the dataset. Adjusting *f*_*o*_ could help in this regard (Golshan et al., 2016, 2018, 2020). In addition, the SVM algorithm does not yield prediction probabilities. Given the importance of thresholding for state transition, those cannot be omitted and thus have to be estimated in an additional *post hoc* process (Platt, 1999). LR and the LDA classifiers may be thus preferable with the LR was more easily calibrated through regularization. Ensemble methods, using a set of classifiers rather than a single unit (in particular decision trees), have proven to outperform other machine-learning methods when deployed on small size, complex, and noisy BCI datasets (Gruenwald et al., 2019; Zhu et al., 2020) and could be easily implemented using the proposed method. The method could also be applicable to neural-network based decoders (Ahmadi et al., 2019; Golshan et al., 2020) specifically tuning of decoder specific parameters such as selected channels, thresholds or window convolution size.

## 5. Conclusion

We have introduced a decoding architecture based on BO to tune different parameters in the feature extraction, channel selection, classification and state-transition stages of an asynchronous decoder to identify movement signatures from STN-LFPs signals recorded in the deep brain. This architecture allows self-tuning in a computationally efficient implementation for real-time deployment. Although asynchronous decoding from STN-LFPs remains highly subject-specific, we demonstrate that BO improves decoding performance across participants. In addition, BO provided in-depth details regarding the tuning process and the relevance of each parameter which could help inform future decoder design iterations. For asynchronous decoding, time-frequency signal extraction (regardless of the chosen approach) should render smoother feature signals, and state estimators should be robust to spurious activity change. An increased number of features or decoding channels should be budgeted relative to incremental performance gains and model complexity. Optimal individual parameters are essential, especially where substantial inter-subject variability is present, to best adapt to the characteristics of the neural signals. The method presented in this study could also be applied to decoding other bulkier movements, arousal, and pathological states to construct feedback mechanisms for adaptive DBS systems and serve other BCI applications. Future work should include further investigation of other DBS-recorded LFPs signals and further online testing.

## Data availability statement

Publicly available datasets were analyzed in this study. This data can be found here: https://data.mrc.ox.ac.uk/mrcbndu/data-sets/search.

## Ethics statement

The studies involving human participants were reviewed and approved by the South Central – Oxford C Research Ethics Committee 18/SC/0006. The patients/participants provided their written informed consent to participate in this study.

## Author contributions

HT and SH gathered the data used in the computational experiment. TM designed and implemented the computational experiment, analyzed the experimental results, and wrote the manuscript. SH, HT, and RV reviewed and edited the manuscript. All authors contributed to the article and approved the submitted version.
